# ASSESSING THE COMPLEXITIES OF LONG-TERM SERVICES AND SUPPORTS FOR OLDER ADULTS OF TEXAS

**DOI:** 10.1093/geroni/igae098.2446

**Published:** 2024-12-31

**Authors:** Sara Murphy, Esme Anaab, Jennifer Severance, Robert Yockey

**Affiliations:** University of North Texas Health Science Center, Fort Worth, Texas, United States; University of North Texas Health Science Center, Fort Worth, Texas, United States; University of North Texas Health Science Center, Fort Worth, Texas, United States; University of North Texas Health Science Center, Fort Worth, Texas, United States

## Abstract

**Background:**

Long-term services and supports (LTSS) encompass a broad range of medical and personal care assistance needed by individuals who are unable to perform self-care tasks due to aging, chronic illness, or disability. The No Wrong Door (NWD) System is a statewide network that coordinates the system of access to long-term services and supports. As an offshoot of the person-centered planning movement, the NWD System is premised on ensuring that no matter where individuals first interact with the system, they are guaranteed comprehensive information, assessment, and services. This work will help the Texas Health and Human Services Commission create a consumer-driven, cost-effective, and efficient system.

**Methods:**

A mixed methods approach was constructed to understand the challenges and barriers to accessing LTSS, assess NWD System performance based on stakeholder perspectives, and identify community needs.

**Results:**

Of 4,153 respondents, 56% identified as an adult 50 years or older and 53% older adults reported a LTSS need in the past year. The top three problems faced when trying to get services include a confusing system, long service wait times, and not knowing where to get help. Unpaid informal caregiver respite services, caregiver education, and caregiver support groups were among the services and supports that inadequately met people’s needs. Conclusions: Our results form the blueprint for facilitating access to care throughout Texas to ensure vulnerable populations obtain a high quality of life. Recommendations for the NWD system include effective community outreach and streamlined application processes for services and supports.

